# Supplementary material for: Deep and accurate detection of m6A RNA modifications using miCLIP2 and m6Aboost machine learning
Source: Nucleic Acids Res. 2021 Jun 22;49(16):e92. doi: 10.1093/nar/gkab485 (PMC8450095; doi:10.1093/nar/gkab485)
Supplement: gkab485_Supplemental_Files [file gkab485_supplemental_files.zip › Supplementary_Material_v23.pdf]

Deep and accurate detection of m^6A RNA modifications using miCLIP2 and m6Aboost machine learning

Nadine Körtel[1,#], Cornelia Rücklé[1,#], You Zhou[2,#], Anke Busch[1], Peter Hoch-Kraft[1], FX Reymond Sutandy[1,3], Jacob Haase[4], Mihika Pradhan[1], Michael Musheev[1], Dirk Ostareck[5], Antje Ostareck-Lederer[5], Christoph Dieterich[6,7], Stefan Hüttelmaier[4], Christof Niehrs[1,8], Oliver Rausch[9], Dan Dominissini[10], Julian König[1,*], and Kathi Zarnack[2,*]

[1] Institute of Molecular Biology (IMB), Mainz, 55128, Germany. [2] Buchmann Institute for Molecular Life Sciences (BMLS) & Faculty of Biological Sciences, Goethe University Frankfurt, Frankfurt, 60438, Germany. [3] Institute of Biochemistry II, Goethe University Frankfurt, Frankfurt, 60590, Germany. [4] Institute of Molecular Medicine, Sect. Molecular Cell Biology, Martin Luther University Halle-Wittenberg, Charles Tanford Protein Center, Halle, 06120, Germany. [5] Department of Intensive Care Medicine, University Hospital RWTH Aachen, Aachen, 52074, Germany. [6] Klaus Tschira Institute for Integrative Computational Cardiology, University Hospital Heidelberg, Heidelberg, 69120, Germany. [7] German Centre for Cardiovascular Research (DZHK) - Partner Site Heidelberg/Mannheim, Heidelberg, 69120, Germany. [8] Division of Molecular Embryology, DKFZ-ZMBH Alliance, Heidelberg, Germany. [9] STORM Therapeutics Ltd, Cambridge, CB22 3AT, UK. [10] Cancer Research Center and Wohl Institute for Translational Medicine, Chaim Sheba Medical Center, Tel HaShomer, and Sackler School of Medicine, Tel Aviv University, Tel Aviv, 6997801, Israel.

[#] These authors contributed equally. Order of first authors was determined by lottery.
* Corresponding authors: Kathi Zarnack (kathi.zarnack@bmls.de) & Julian König (j.koenig@imb-mainz.de)

SUPPLEMENTARY MATERIAL

Content:

Supplementary Methods 2
A. Differential methylation analysis 2
B. AdaBoost machine learning to identify true m^6A sites 4
Supplementary Figures 7
Supplementary Tables 16
Supplementary References 19

Supplementary Methods

A. Differential methylation analysis

In order to discriminate true m^6A sites from background in the miCLIP2 data, we compared miCLIP2 profiles from wildtype (WT) and *Mettl3* knockout (KO) mouse embryonic stem cells (mESCs) which deplete m^6A modifications from mRNAs (**Figure 1B**). However, this analysis was confounded by broad changes in gene expression in response to the *Mettl3* KO, which resulted in 5,372 differentially regulated genes including 3,005 up- and 2,367 down-regulated genes (false discovery rate [FDR] ≤ 0.01; **Figure 2A**). Since the miCLIP2 signal, similar like regular iCLIP (1,2), is strongly dependent on the underlying transcript abundance (**Supplementary Figure S2B**, top panel), this means that if not corrected for, differential analysis will erroneously pick up many peaks with reduced signal in downregulated genes. In order to illustrate this, we applied DESeq2 (3) collectively to all peaks in the dataset (approach termed *one-run*) which thus tests each peak independently and omits the underlying transcript level changes. These and all following analyses are based on miCLIP2 truncation reads in peaks identified by peak calling with PureCLIP (4) (see Methods). As expected, the changes in transcript abundance were mirrored in estimated fold changes in miCLIP2 signal of the associated peaks, such that almost all peaks in strongly downregulated genes (log2FC < -3.5) went down in the *Mettl3* KO, whereas peaks in upregulated genes tended to go up (**Supplementary Figure S3A**, left).

In order to overcome this, we tested three different approaches based on (i) separately running DESeq2 on the peaks of each gene (*gene-wise*), (ii) combining peaks for groups of genes with similar abundance change (*bin-based*), and (iii) adopting DEXSeq (*dexseq-run*) (5) instead of DESeq2 (**Supplementary Figure S3A**). The three approaches worked as follows:

<u>*Gene-wise approach.*</u> Here, we ran an individual DESeq2 analysis for each gene. To this end, we first assigned all peaks to their host gene based on GENCODE gene annotation (release M23) (6). Overlapping genes were resolved by the genes' support level and length, prioritising better support and longer genes. All peaks of a given gene were then used for a collective DESeq2 analysis.

<u>*Bin-based approach.*</u> A disadvantage of testing for individual genes, as in the *gene-wise* approach and in *dexseq-run* below, is that it relies on a sufficient number of peaks per gene to estimate the required parameters, such as the dispersion, correctly. In order to overcome this, we stratified all genes based on their expression changes upon *Mettl3* KO into equally sized bins. We then collectively tested the signal changes of the peaks from all genes within each bin using DESeq2. Gene expression changes were calculated on the total number of miCLIP2 truncation reads in each gene, acquired by htseq-count with default parameters (7) using GENCODE

gene annotation. Comparison with a parallel DESeq2 analysis on matching RNA-seq data for the same samples showed a high correlation of log2FC values (**Supplementary Figure S3B**), supporting that the summed miCLIP2 signal on the genes allows for a reliable estimation of gene expression changes. We next used the miCLIP2-derived log2FC values to stratify all genes into equal bins (width of Δlog2FC = 0.3, 47 bins; **Supplementary Figure S3C**). Subsequently, we applied DESeq2 collectively to all peaks from all genes of the same bin.

<u>*2-factor approach.*</u> In this approach, we built a combined DESeq2 model on the miCLIP2 signal in the peaks and the summed miCLIP2 signal on the genes as a proxy for gene expression. To account for gene expression changes, we tested for an interaction term for the peak signals and gene counts (design = ~condition + condition:geneExpression) to the design formula of the DESeq2 model.

<u>*Dexseq-run.*</u> We adopted DEXSeq (5) (version 1.36.0), an R/Bioconductor package that was developed to test for alternative splicing in RNA-seq data. Originally, DEXSeq2 models RNA-seq read counts in exonic bins, which are grouped by genes, in a generalised linear model to test for differential exon abundance. Additional parameters in the model account for congruent changes across the exons of the same gene to estimate changes in overall gene expression. To run DEXSeq on the miCLIP2 data, we treated each peak as an exonic bin and grouped them by their assigned host gene. DEXSeq was then run with the formula "~sample + peak + condition:peak".

We benchmarked the performance of the different approaches based on the assumptions that true m^6A sites should reside at A and show reduced miCLIP2 signal in the *Mettl3* KO cells. To this end, we compared the differential peaks identified with increasing stringency (FDR) with respect to the total number (yield) and fraction (precision) at A (**Figure 2B**). With more stringent FDR thresholds, the proportion of significantly differential peaks at A continuously increased for all approaches. The best performance was seen for the *bin-based* approach, which yielded the highest number of significant peaks with the highest proportion at A at most FDR thresholds (**Figure 2B**). Besides its accuracy, the *bin-based* approach also showed the best run-time performance by consuming just 1% of CPU time compared to the *gene-wise* or *dexseq-run* approaches.

With the *bin-based* approach at a threshold of FDR < 0.01, we identified total of 14,282 significantly differential peaks, out of which 13,912 peaks (97.4%) went down upon *Mettl3* KO (**Figure 2C**). 11,862 (85.3%) of the decreased peaks resided at A (**Figure 2D**). These were further filtered for the following analyses as described below.

B. AdaBoost machine learning to identify true m^{6}A sites

Compiling the positive and negative sets

We next sought to build a machine learning classifier to distinguish true m^{6}A signals from background in any miCLIP2 datasets, without accompanying *Mettl3* KO. Starting from the reduced peaks at A from the differential methylation analysis (*bin-based* approach, FDR $\leq$ 0.01, log2FC < 0), we calculated the frequency of pentamer motifs at the putative modification site and removed 155 peaks (1.3%) with the most rarely occurring pentamers (present in less than four peaks). This yielded a stringent positive set of 11,707 peaks that were treated as true m^{6}A sites, including 10,966 at DRACH (93.7%) and 741 at non-DRACH motifs (6.3%).

For the corresponding negative set, we required that peaks were not depleted and did not show significant regulation upon the *Mettl3* KO and hence are likely to be part of the nonspecific background in the miCLIP2 data. Based on the *bin-based* approach, we filtered for peaks at A with log2FC ≥ 0 and FDR > 0.5. This yielded a negative set of 42,090 peaks, including 1,043 at DRACH (2.5%) and 41,047 at non-DRACH motifs (97.5%). The unbalanced ratio of roughly 4:1 between the negative and the positive set reflected the observed contribution of true m^{6}A sites among all miCLIP2 peaks (**Figure 4A**).

We combined the positive and negative set and then randomly selected 80% of the sites as our training set. The remaining 20% of the sites were kept as an independent test set.

Feature selection

For all peaks in the training and test set, we extracted the following positional and experimental features:

(i) Surrounding nucleotide sequence: We used a 21-nt window around the putatively modified A nucleotide. This is less than what is used by most existing algorithms that predict m^{6}A sites solely on sequence information, including the popular tools SRAMP and DeepM6ASeq (8-10).

(ii) Transcript region: Since m^{6}A sites accumulate in certain transcript regions (11,12) (**Figure 2F**), we included their location within 5’ UTR, CDS and 3’ UTR as features for the prediction. Transcript annotations were taken from GENCODE (release M23) and filtered for a transcript support level ≤ 3 and support level ≤ 2. Since the same position can residue in different regions of different isoforms, we separately extracted whether a peak overlapped with at least one 5’ UTR, CDS and 3’ UTR, and then used this information as three features for the prediction.

(iii) Relative signal strength: In our initial characterisation of the miCLIP2 data, we found that the relative signal strength offered means to enrich for putative m^{6}A sites (**Supplementary Figure S2C**). The relative signal strength is calculated as the number

of truncation events in each peak divided by the mean number of truncation events for all peaks in the same gene (see Methods). For the prediction, values were increased by a pseudo-count of 1 and then $\log_2$-transformed.

(iv) C-to-T transitions: It was previously shown that in the case of readthrough, C-to-T transitions appear at the sites of m^6A modifications (13). We therefore included the number of C-to-T transitions 1 nt downstream of each peak as orthogonal feature from the miCLIP2 data. The values were increased by a pseudo-count of 1 and then $\log_2$-transformed.

The importance of the features in the final m6Aboost model is shown in **Figure 3B**.

Machine learning approaches tested for m^6A site prediction from miCLIP2 data

We initially tested three different machine learning algorithms. (i) AdaBoost: Adaptive boosting (AdaBoost) formulated by Yoav Freund and Robert Schapire (14) is one of the most widely known boosting algorithms. This tree boosting algorithm combines decision stumps (weak learners) and turns them into a strong learner via applying the boosting method. Moreover, AdaBoost performs exceptionally well for dichotomous tasks. In this project, we used the R package adabag (15) to construct the AdaBoost-based m^6A predictor m6Aboost. (ii) Support vector machine (SVM) is a popular machine learning algorithm in bioinformatics which transfers the data to a higher dimension and then finds the hyperplanes to best classify the samples. It was previously used for the prediction of mammalian m^6A modification sites (16,17). In this project, we used an interface (e1071) of LIBSVM (18) in the R language to construct the SVM-based predictor. (iii) Random Forest (RF) is a decision tree-based algorithm which shows an excellent performance in supervised learning. It is used in SRAMP (8), one of the earliest and most commonly used m^6A predictors. We used an R package of randomForest (19) to build the RF-based m^6A predictor.

For all three classifiers, we evaluated the prediction performance on the independent test set using precision-recall (PR) curves (**Figure 3C and Supplementary Figure S4B**). We also calculated sensitivity, specificity, accuracy, precision, F1-score and Matthews correlation coefficient (MCC) (**Supplementary Figure S4A**) as follows:

$$Sensitivity = Recall = \frac{TP}{TP+FN} \quad (1)$$

$$Specificity = \frac{TN}{TN+FP} \quad (2)$$

$$Accuracy = \frac{TP+TN}{TP+TN+FP+FN} \quad (3)$$

$$Precision = \frac{TP}{TP+FP} \quad (4)$$

$$F1 = 2 * \frac{Precision * Recall}{Precision+Recall} \quad (5)$$

$$MCC = \frac{TP * TN - FP * FN}{\sqrt{(TP+FP)(TP+FN)(TN+FP)(TN+FN)}} \quad (6)$$

where TP, TN, FP and FN represent the counts of true positive, true negative, false positive and false negative predictions, respectively. For m6Aboost, we additionally employed 5-fold cross-validation using the area under the curve (AUC) of PR curves and receiver operating characteristic (ROC) curves to measure the prediction performance (**Supplementary Figure S4C**). We also tested a variant of the AdaBoost model that was trained and tested on a balanced setup (**Supplementary Figure S4A**). For this, we randomly subsampled the negative set to 11,707 sites to match the positive set. Based on the employed measures, we selected the AdaBoost-based predictor m6Aboost (**Supplementary Figure S4D**).

Normalisation of numerical features

Application of the machine learning model to new datasets requires that the data were generated by the same protocol and thus show an independent and identical distribution. The m6Aboost model includes two numerical features from the miCLIP2 data, namely relative signal strength and C-to-T transitions, which could systematically vary between experiments. Since in the training set, both features approximated a Poisson distribution (**Supplementary Figure S4E**), we normalised the values of each features in the input samples by the ratio of the mean for this feature between the input dataset and the training set.

Supplementary Figures

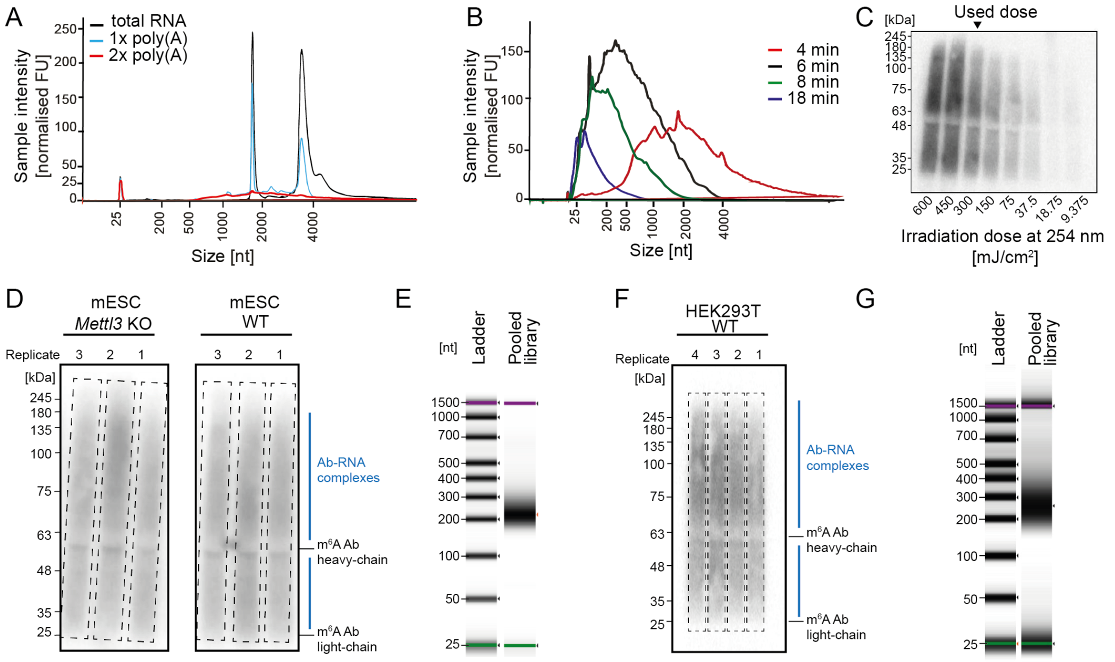

Supplementary Figure S1. miCLIP2 library preparation. *A-C.* Optimisation of the miCLIP2 protocol. ***A.*** Ribosomal RNA is fully depleted after two rounds of poly(A) selection. Electropherogram illustrating poly(A) enrichment for 1 µg total RNA of HEK293T cells after one (1x, blue) and two (2x, red) rounds of poly(A) selection. ***B.*** An incubation time of 8 min results in optimal RNA fragmentation. For an optimal RNA fragment spectrum between 50-200 nt, fragmentation periods of 4-18 minutes were compared. ***C.*** Autoradiograph illustrating comparison of RNA crosslinked to the m^{6}A antibody at different irradiation doses in mJ/cm² at 254 nm UV light. The expected molecular weight of the m^{6}A antibody is 50 kDa. ***D-G.*** Visualisation of the miCLIP2 libraries from mouse embryonic stem cells (mESCs) (D,E) and human HEK293T cells (F,G). ***D.*** Autoradiograph illustrating radioactively labelled m^{6}A-antibody-RNA complexes from wild-type (WT) and *Mettl3* knockout (KO) mESCs. The expected molecular weight of the anti-m^{6}A antibody is 50 kDa for the heavy-chain and 25 kDa for the light chain. Excised regions are indicated with dotted lines. ***E.*** Final miCLIP2 pooled library of three biological replicates each for mESC WT and mESC *Mettl3* KO. Note that the final library contained independent samples from an unrelated experiment that were multiplexed for high-throughput sequencing. ***F.*** Autoradiograph illustrating radioactively labelled m^{6}A-antibody-RNA complexes from HEK293T cells. Excised regions are indicated with dotted lines. ***G.*** Final miCLIP2 pooled library of four biological replicates from HEK293T cells. Note that the pooled library also includes material from an additional experiment which is not part of this study.

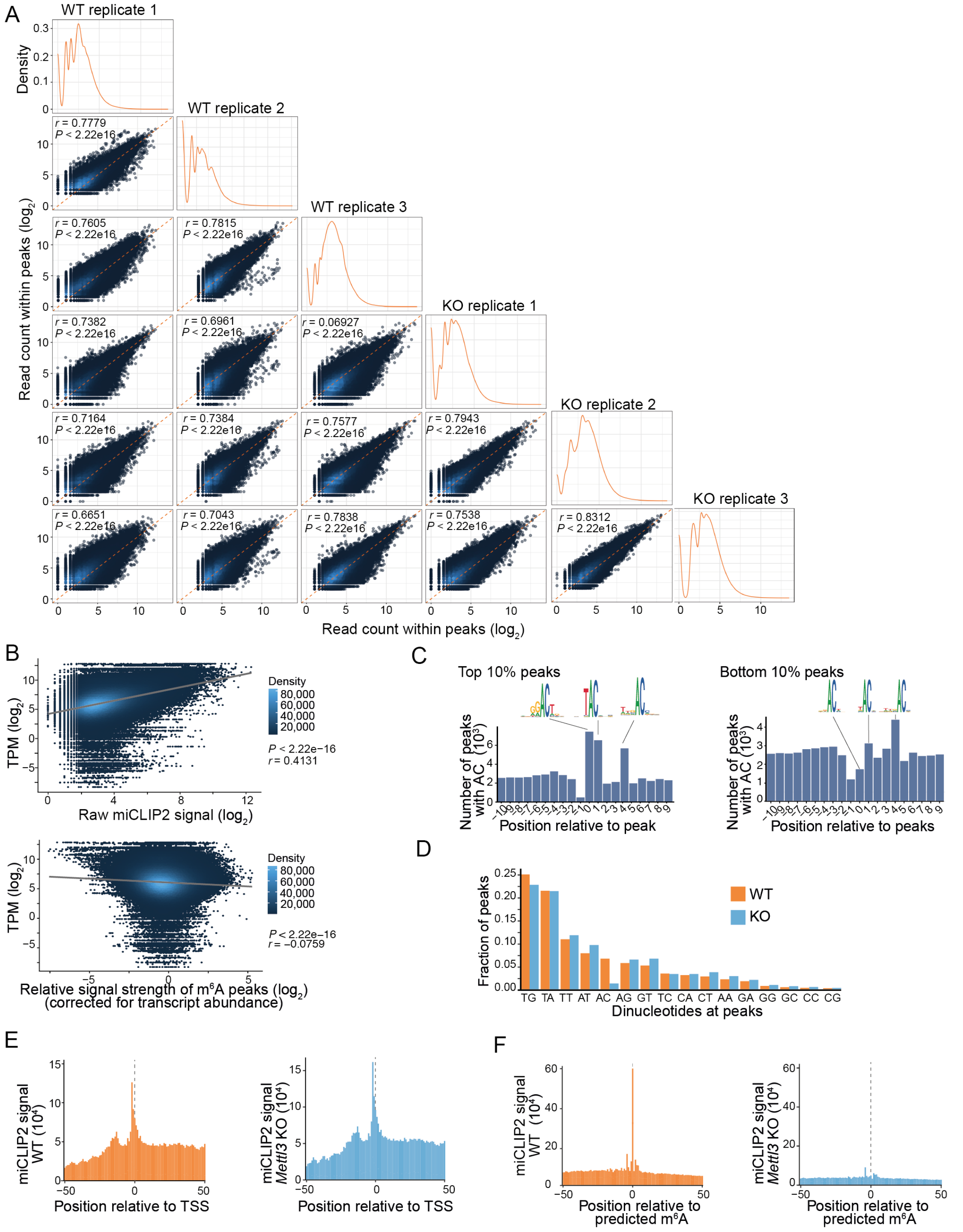

Supplementary Figure S2. Data quality. ***A.*** miCLIP2 libraries are highly reproducible between replicates. Pairwise comparison of truncation read counts within peaks for all replicates from WT and *Mettl3* KO mESCs are shown as an extension of **Figure 1C**. Pearson correlation coefficients (*r*) and associated *P* values are given. ***B.*** Relative signal strength corrects for the effect of gene expression on the miCLIP2 signal. Scatter plots show correlation between miCLIP2 truncation reads (top) or relative signal strength (bottom) and expression of the respective gene (in transcripts per million, TPM, log_2) for all peaks from the WT miCLIP2 data. Colour gradient shows point density. Pearson correlation coefficients (*r*) and associated *P* values are given. ***C.*** Stronger peaks are more often located at AC dinucleotides than weaker peaks. AC

dinucleotide content in a 21-nt window around the 10% strongest peaks (relative signal strength) compared to the 10% weakest peaks from miCLIP2 WT data. ***D.*** Less peaks are located at AC dinucleotides in the *Mettl3* KO miCLIP2 data. Dinucleotide distribution of all peaks from the miCLIP2 WT (orange) and *Mettl3* KO (blue) experiment. ***E.*** Transcript start sites (TSS) accumulate miCLIP2 WT signal which is not reduced upon *Mettl3* KO. miCLIP2 truncation events in a 101-nt window relative to the TSS from WT (upper, orange) and *Mettl3* KO (lower, blue) data. ***F.*** miCLIP2 WT signal enriches around predicted m^6A sites and is depleted in the *Mettl3* KO. miCLIP2 truncation events in a 101-nt window relative to the predicted m^6A sites from WT (upper, orange) and *Mettl3* KO (lower, blue) data.

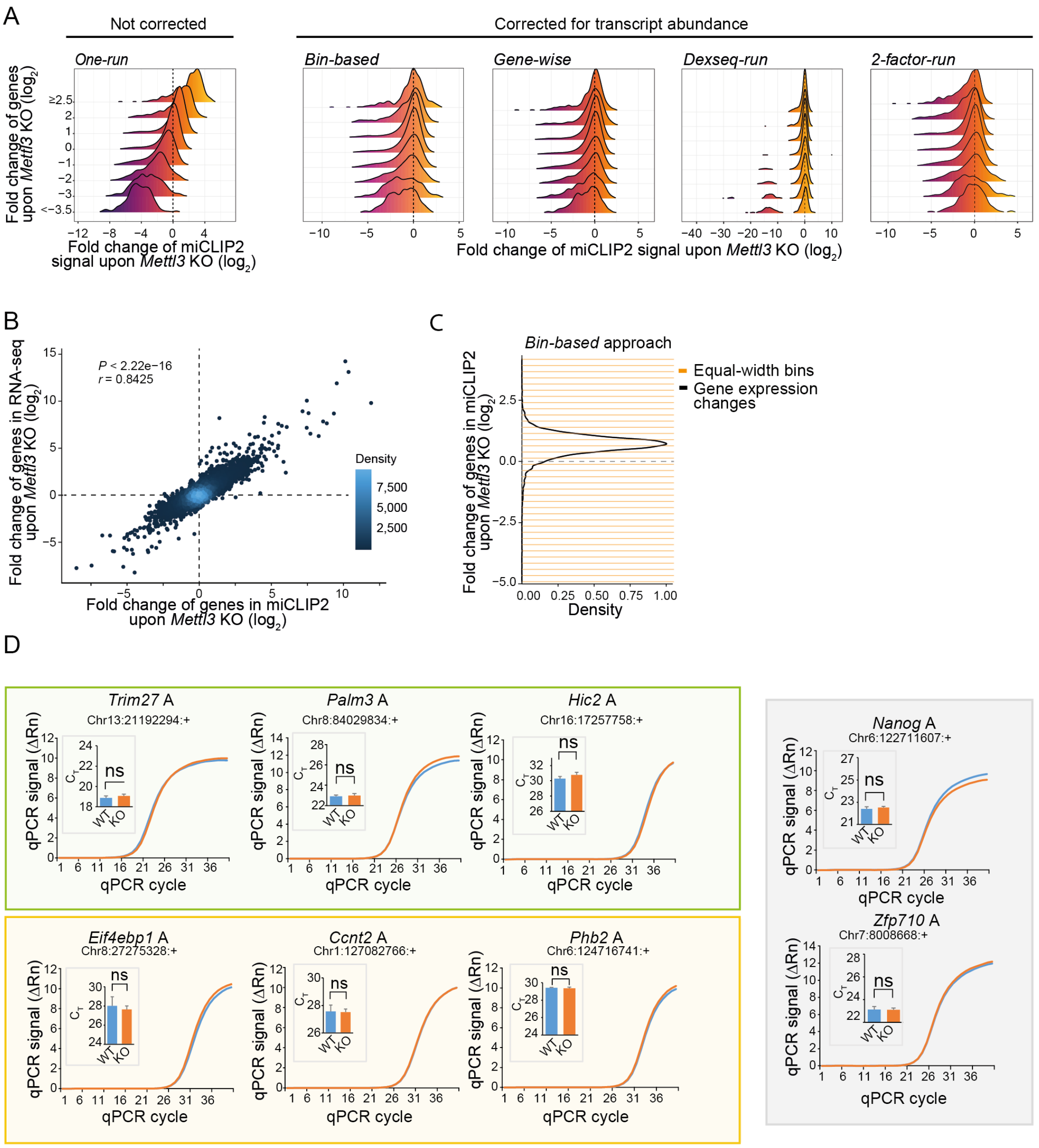

Supplementary Figure S3. Differential methylation analysis and validation. ***A.*** The *bin-based* approach allows to correct for expression changes between WT and *Mettl3* KO. For each tested differential methylation method, the fold change of genes upon *Mettl3* KO (y-axis) and the estimated fold change in the miCLIP2 signal upon the *Mettl3* KO (x-axis) are compared. The *one-run* approach, which does not correct for gene expression changes, is shown for comparison. ***B.*** Changes in gene expression can be estimated from the miCLIP2 data. Comparison of fold changes of genes (n=14,989) calculated from the miCLIP2 data (x-axis) and matching RNA-seq data (y-axis). Pearson correlation coefficients (*r*) and associated *P* value are given. ***C.*** For the *bin-based* approach, genes were stratified based on their expression changes upon *Mettl3* KO (log_2-transformed fold change) into equal-width bins (width of Δlog2FC = 0.3, highlighted in orange). The peaks on all genes within each bin were then collectively tested for differential methylation. ***D.*** Complemental control positions of unmodified A sites neighbouring the validates m^6A sites at non-DRACH (olive background) and DRACH (orange background) motifs (**Figure 2H and I**) as well

as two unmodified sites (grey) at a DRACH motif (**Figure 2J**). Exemplary real-time fluorescence amplification curves (normalised reporter value, ΔRn) and quantification of threshold cycle (C_T) values (technical replicates) for SELECT experiments with mESC WT versus *Mettl3* KO samples are shown for neighbouring unmodified A nucleotides (gene name and genomic coordinates given above). ns, not significant, two-sided Student's *t*-test, n=3.

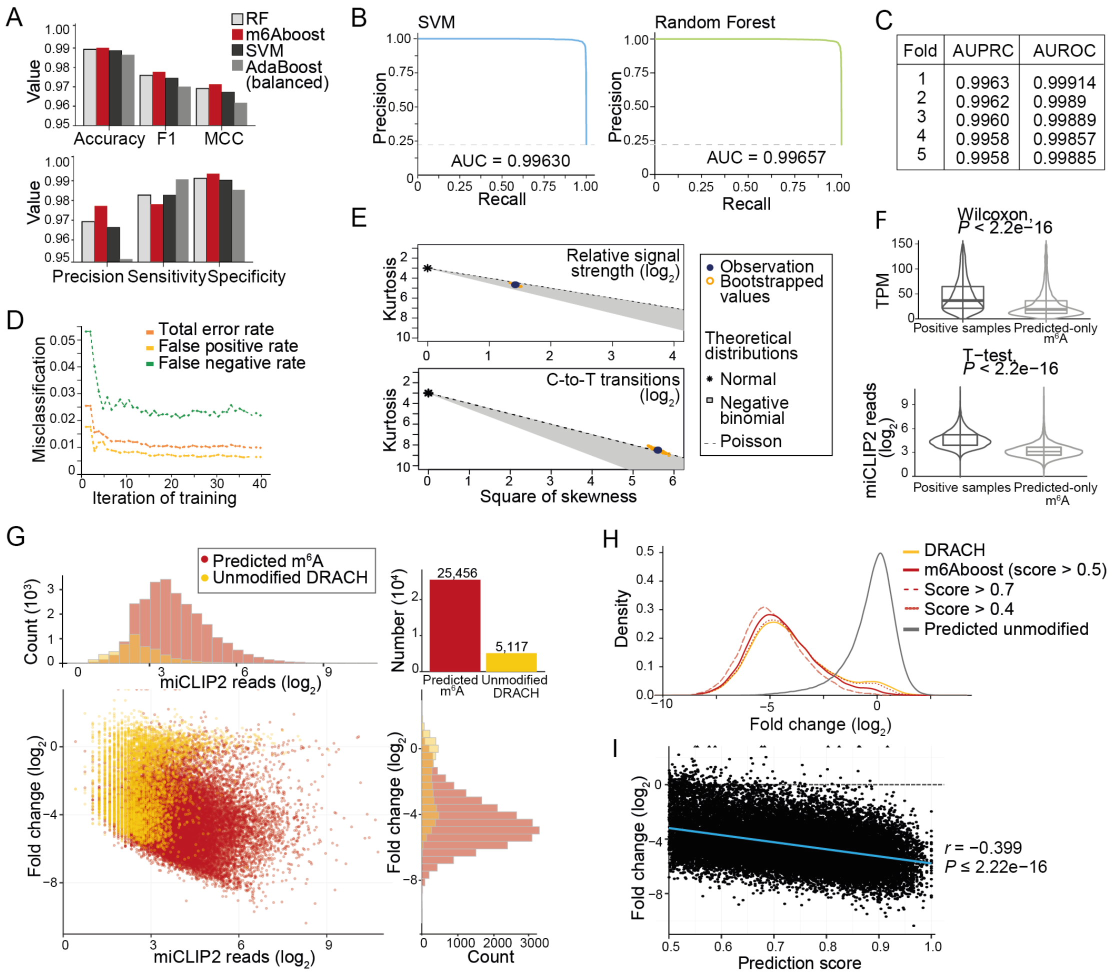

Supplementary Figure S4. Machine learning to predict m^{6}A sites from miCLIP2 data. ***A.*** Performance measures to compare four different machine learning models. Accuracy, F1-score, Matthews correlation coefficient (MCC), precision, sensitivity and specificity (see **Supplementary Material**) are given for models based on AdaBoost (m6Aboost), support vector machine (SVM) and random forest (RF). An AdaBoost models trained on a balanced test set is shown for comparison. ***B.*** Precision-recall curves for the support vector machine (SVM; left) and random forest (right) models. PR curve for m6Aboost is shown in **Figure 3C**. The corresponding area under the curve (AUC) is given. ***C.*** Results of five-fold cross-validation for m6Aboost. AUC are given for receiver operating characteristic (AUROC) and precision recall curve (AUPRC). ***D.*** Progressive training of m6Aboost. Graph displays misclassification (y-axis) per iteration of training (x-axis). Total error rate (orange), false positive rate (yellow) and false negative rate (green) are shown. ***E.*** Log$_2$-transformed relative signal strength values (top) and C-to-T transitions per peak follow a Poisson distribution. Cullen and Frey graphs compare square of skewness (x-axis) against kurtosis (y-axis) of the two experimental features (observation and 100 bootstrapped values) against normal, negative binominal and Poisson distribution. ***F.*** m^{6}A sites that are predicted by m6Aboost but not part of the positive set preferentially occur in lowly expressed genes. Top, boxplot shows gene expression values (in transcripts per million, TPM) for 4,292 genes with m^{6}A sites from the positive set against 5,640 genes which exclusively harbour m^{6}A sites that

were only predicted by m6Aboost. *P* value < 2.2e-16, Wilcoxon rank-sum test. Bottom, boxplot compares number of miCLIP2 reads (log_2-transformed) in 11,707 m^6A sites from the positives set against 13,908 m^6A sites that were only predicted by m6Aboost. *P* value < 2.2e-16, Student's *t*-test. ***G.*** Scatter plot and associated histograms show fold change in miCLIP2 signal (log_2-transformed, x-axis) against number of miCLIP2 reads per peak (log_2-transformed, y-axis) for 5,117 peaks at DRACH motifs (yellow) that are predicted to be unmodified by m6Aboost. m^6A sites predicted by m6Aboost (red) are shown for comparison. ***H.*** m6Aboost associates a probability with each predicted m^6A site which can be used to filter more stringently. Density of predicted m6A sites identified with various probability scores against the log_2-transformed fold change between WT and *Mettl3* KO of the corresponding sites. Results of different prediction scores are shown (score [s] = 0.5, red, s=0.7, dashed, s=0.4, dotted) and filtering for a DRACH motif only (orange), as well as the distribution of unmodified sites. ***I.*** The m6Aboost prediction score correlates with the change in miCLIP2 signal upon *Mettl3* KO. Scatterplot showing log_2-transformed fold change in miCLIP2 read counts in WT versus *Mettl3* KO mESC (y-axis) against m6Aboost prediction score (x-axis).

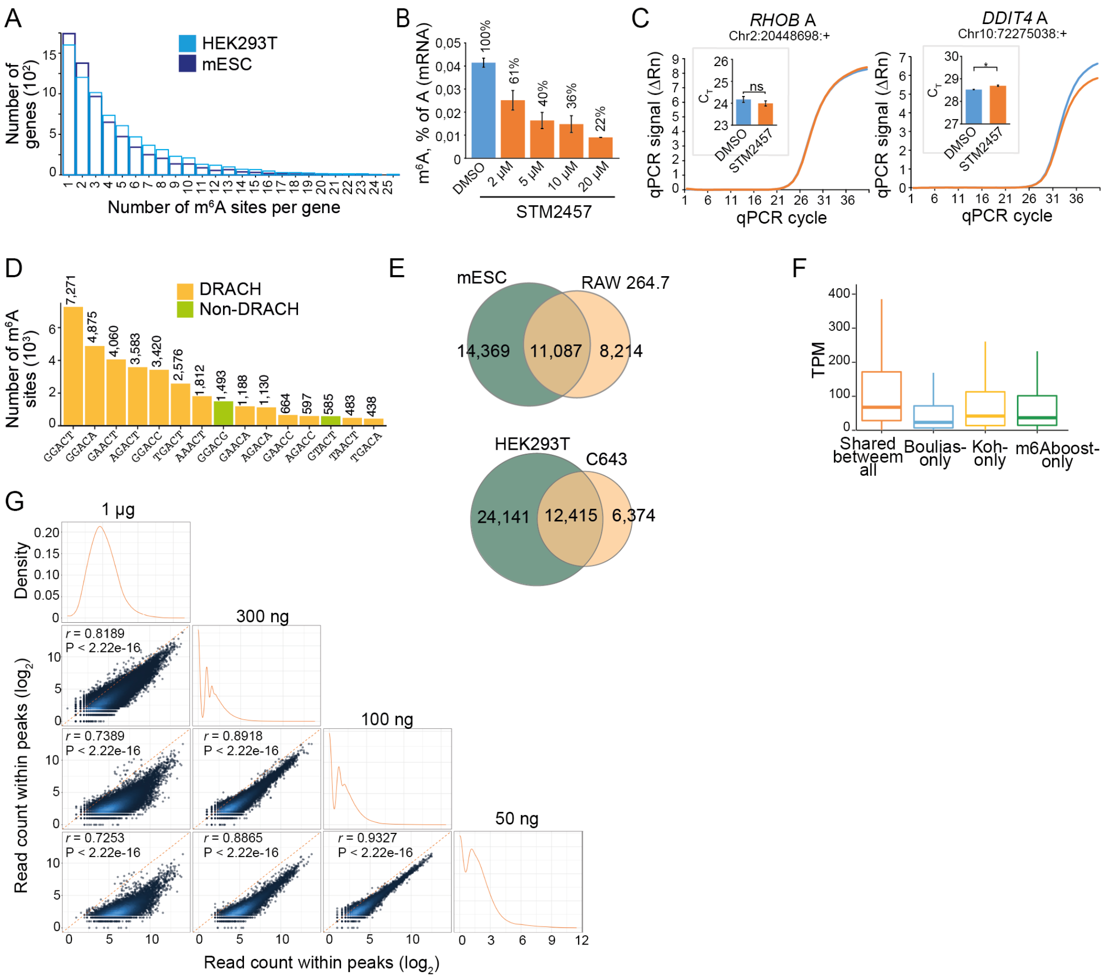

Supplementary Figure S5. m^{6}A sites predicted in miCLIP2 data from human HEK293T and mouse heart tissue. ***A.*** Most genes carry up to three m^{6}A sites. Barplot shows the number of genes (y-axis) with the given number of m^{6}A sites (x-axis) in the miCLIP data from mESC WT (orange) and HEK293T (blue) cells. ***B.*** Titration with increasing concentrations of the METTL3 inhibitor (STM2457)(20) on HEK293T cells shows a gradual reduction of m^{6}A levels on mRNAs. m^{6}A levels measured by liquid chromatography-tandem mass spectrometry (LC-MS/MS) for poly(A)+ RNA from untreated (DMSO) and 2 µM, 5 µM, 10 µM and 20 µM STM2457-treated HEK293T cells. Quantification of m^{6}A as percent of A in RNA. Error bars indicate standard deviation of mean (s.d.m.), n = 3. ***C.*** Complementary control positions of unmodified A sites neighbouring the validates m^{6}A sites from HEK293T cells in the genes *RHOB* and *DDIT4* (**Figure 5B**). Exemplary real-time fluorescence amplification curves (normalised reporter value, ΔRn) and quantification of threshold cycle (C_T) values (technical replicates) for SELECT experiments with untreated (DMSO) versus STM2457-treated samples are shown for neighbouring unmodified A nucleotides (gene name and genomic coordinates given above). ns, not significant, two-sided Student's *t*-test, n=3. ***D.*** The most frequent pentamers at m6Aboost-predicted m^{6}A sites in HEK293T include DRACH and non-DRACH motifs. Same as **Figure 2G** for predicted m^{6}A sites from HEK293T cells. ***E.*** About half of all m^{6}A sites are shared between the two cell lines. Venn diagram as **Figure 4E** (mouse) and **Figure 5H** (human) for m^{6}A sites on all genes (not filtered for expression). ***F.*** m^{6}A sites that

were only found in one out of three datasets from HEK293T cells (this study, (21,22)) are located in lowly expressed genes. Boxplot summarises expression of genes (in transcripts per million, TPM, log_2) harbouring m^6A sites unique to one dataset. ***G.*** miCLIP2 data from different amounts of RNA input are reproducible. Pairwise comparison of truncation read counts within peaks for miCLIP2 libraries from decreasing amounts of RNA input material from mouse heart tissue. Pearson correlation coefficients (*r*) and associated *P* values are given.

Supplementary Tables

Supplementary Table S1. Summary of miCLIP2 experiments. Table includes information on all conducted miCLIP2 experiments including sample names, cell or tissue type, and employed barcodes. It further specifies number of uniquely mapped reads, how many of those were truncation reads (no C-to-T transition) or harboured C-to-T transitions. For each condition, the number of identified PureCLIP peaks and m6Aboost-predicted m^6A sites are given. [provided as Excel file]

Supplementary Table S2. Oligonucleotides used in SELECT experiments in **Figure 2H-J and 5B and Supplementary Figure S3D and S5C**. qPCR oligonucleotides for target genes were used for normalisation of input material. Names indicate target and position relative to targeted m^6A site. Oligonucleotides were designed complementary anneal to RNA leaving a gap at targeted m^6A site or adjacent A site (UP and DOWN probe). Lowercase letters represent adapter sequences for qPCR as described in (23). Uppercase letters represent complementary sequence to target site. Phos indicates 5' phosphorylation.

Name	Sequence [5' - 3']
qPCR_fwd for SELECT	ATGCAGCGACTCAGCCTCTG
qPCR_rev for SELECT	TAGCCAGTACCGTAGTGCGTG
m^6A sites from mESC:	
Eif4ebp1_qPCR_fwd	ACTCACCTGTGGCCAAAACA
Eif4ebp1_qPCR_rev	TTGTGACTCTTCACCGCCT
Eif4ebp1_m^6A_UP	tagccagtaccgtagtgcgtgGGGAGGGTGTGAGTGAGA G
Eif4ebp1-m^6A_DOWN	[Phos]CATTCCCCTGCAGTAGCAGcagaggctgagtcgctgcat
Eif4ebp1_m^6A-4 UP	tagccagtaccgtagtgcgtgGGGTGTGAGTGAGAGTCAT
Eif4ebp1_m^6A-4 DOWN	[Phos]CCCCTGCAGTAGCAGCTCGcagaggctgagtcgctgcat
Ccnt2_qPCR_fwd	GGGCAACGTCTCAATGTCTCT
Ccnt2_qPCR_rev	AAGCTTTCGAGCCTGCTCTT
Ccnt2_m^6A_UP	tagccagtaccgtagtgcgtgGCCCATGCTTGTGCTGCTG
Ccnt2_m^6A_DOWN	[Phos]TCTGCATGGGCAGCTAGATcagaggctgagtcgctgcat
Ccnt2_m^6A+2_UP	tagccagtaccgtagtgcgtgCGGCCCATGCTTGTGCTGC
Ccnt2_m^6A+2_DOWN	[Phos]GTTCTGCATGGGCAGCTAGcagaggctgagtcgctgcat
Phb2_qPCR_fwd	ATCCGTGTTCACCGTGGAAG
Phb2_qPCR_rev	ACCAGGGGATCCTGAAGTGA
Phb2_m^6A_UP	tagccagtaccgtagtgcgtgGAGGGCAGATACAGAAAAG
Phb2_m^6A_DOWN	[Phos]CCATCACATGATGCCTGGGcagaggctgagtcgctgcat
Phb2_m^6A-4_UP	tagccagtaccgtagtgcgtgGCAGATACAGAAAAGTCCA
Phb2_m^6A-4_DOWN	[Phos]CACATGATGCCTGGGGCAGcagaggctgagtcgctgcat
Trim27_qPCR_fwd	GGAGGGCTTCAAGGAGCAAA
Trim27_qPCR_rev	AGCTGCTCAAACTCCCAGAC
Trim27_m^6A_UP	tagccagtaccgtagtgcgtgACAATGACACTGCCCAGAA
Trim27_m^6A_DOWN	[Phos]CCATTCTGGGGGGCTGAGGcagaggctgagtcgctgcat
Trim27_m^6A-4_UP	tagccagtaccgtagtgcgtgTGACACTGCCCAGAATCCA
Trim27_m^6A-4_DOWN	[Phos]TCTGGGGGGCTGAGGTCACcagaggctgagtcgctgcat

[continued on next page]

Supplementary Table S2. Oligonucleotides used in SELECT experiments (continued from previous page).

Name	Sequence [5' - 3']
Palm3_qPCR_fwd	TACAGCTGTTGCAAAGTGCG
Palm3_qPCR_rev	CACATCAGTCGGGGCGGTA
Palm3_m^{6}A_UP	tagccagtaccgtagtgcgtgTGGGGACCCTCTCGCTCAG
Palm3_m^{6}A_DOWN	[Phos]ACAGGGCTCAGGCTTACTGcagaggctgagtcgctgcat
Palm3_m6A-8_UP	tagccagtaccgtagtgcgtgCTCTCGCTCAGTACAGGGC
Palm3_m6A-8_DOWN	[Phos]CAGGCTTACTGGCTGCCCCcagaggctgagtcgctgcat
Hic2_qPCR_fwd	CTGGCAGGCACCTGAGGTAA
Hic2_qPCR_rev	AGCTGTAGCAGGAGCTGTTT
Hic2_m^{6}A_UP	tagccagtaccgtagtgcgtgTGCCAGCAGTACCCACTCG
Hic2_m^{6}A_DOWN	[Phos]CCAGGGCCAAAGGGCTTGCcagaggctgagtcgctgcat
Hic2_m^{6}A+3_UP	tagccagtaccgtagtgcgtgCTATGCCAGCAGTACCCAC
Hic2_m^{6}A+3_DOWN	[Phos]CGTCCAGGGCCAAAGGGCTcagaggctgagtcgctgcat
Unmodified DRACH sites:	
Nanog_qPCR_fwd	ACCTGAGCTATAAGCAGGTTAAGAC
Nanog_qPCR_rev	CCCTGGGGATAGCTGCAATG
Nanog_nom^{6}A_UP	tagccagtaccgtagtgcgtgCAGGACTTGAGAGCTTTTG
Nanog_nom^{6}A_DOWN	[Phos]TTGGGACTGGTAGAAGAATcagaggctgagtcgctgcat
Nanog_nom^{6}A+2_UP	tagccagtaccgtagtgcgtgCTCAGGACTTGAGAGCTTT
Nanog_nom^{6}A+2_DOWN	[Phos]GTTTGGGACTGGTAGAAGAacagaggctgagtcgctgcat
Zfp710_qPCR_fwd	TACCGCAGCCAGCTACAAAA
Zfp710_qPCR_rev	CTCCTTCACACCCTTGTGGG
Zfp710_nom^{6}A_UP	tagccagtaccgtagtgcgtgGTTTGCTTCTGCACGAAGG
Zfp710_nom^{6}A_DOWN	[Phos]CTTGAAGCAGATGTGGCACcagaggctgagtcgctgcat
Zfp710_nom6A-3_UP	tagccagtaccgtagtgcgtgTGCTTCTGCACGAAGGTCT
Zfp710_nom6A-3_DOWN	[Phos]GAA GCA GAT GTG GCA CTG Gcagaggctgagtcgctgcat
m^{6}A sites from HEK293T:	
DDIT4_qPCR_fwd	TCGTCGTCCACCTCCTCTTC
DDIT4_qPCR_rev	GGTAAGCCGTGTCTTCCTCC
DDIT4_m6A_UP	tagccagtaccgtagtgcgtgCTTGGGCCAGAGTCGTGAG
DDIT4_m6A_DOWN	[Phos]CCAGGCGCAGCACGAGGGTcagaggctgagtcgctgcat
DDIT4_m6A+4_UP	tagccagtaccgtagtgcgtgGGATCTTGGGCCAGAGTCG
DDIT4_m6A+4_DOWN	[Phos]GAGTCCAGGCGCAGCACGAcagaggctgagtcgctgcat
RHOB_qPCR_fwd	CAGTAAGGACGAGTTCCCCG
RHOB_qPCR_rev	GTCCACCGAGAAGCACATGA
RHOB_m6A_UP	tagccagtaccgtagtgcgtgAAGCTGTGTCCTCCCCAAG
RHOB_m6A_DOWN	[Phos]CAGTTGCAAATGTCTTCCCcagaggctgagtcgctgcat
RHOB_m6A-4_UP	tagccagtaccgtagtgcgtgTGTGTCCTCCCCAAGTCAG
RHOB_m6A-4_DOWN	[Phos]TGCAAATGTCTTCCCCAGGcagaggctgagtcgctgcat
Not validated site:	
ABT1_qPCR_fwd	AAGAAACGGGTAGTGCCAGG
ABT1_qPCR_rev	GTCTCACGAACCGGTCCTC
ABT1_m6A_UP	tagccagtaccgtagtgcgtgAGTCCCTGACAAGGGAAGG
ABT1_m6A_DOWN	CCCTCCATGCTCTCTGAGGcagaggctgagtcgctgcat
ABT1_m6A-4_UP	tagccagtaccgtagtgcgtgCCTGACAAGGGAAGGTCCC
ABT1_m6A-4_DOWN	CCATGCTCTCTGAGGGTGGccagaggctgagtcgctgcat

Supplementary Table S3. Overlap of predicted m^6A sites and SCARLET-validated sites in HEK293T cells taken from (24). m^6A sites with >5% modification are shown in bold. Genomic coordinates are relative to human genome version GRCh38.p13.

Genomic coordinate	Motif	Percent methylation according to SCARLET	Predicted by m6Aboost for HEK293T miCLIP2
**Chr11:65500276**	**GGACU**	**0.41**	**yes**
**Chr11:65500338**	**GGACU**	**0.51**	**yes**
**Chr11:65500372**	**GGACU**	**0.13**	**yes**
Chr11:65500435	AGACU	0.03	no
Chr11:65500445	AGACA	0.02	no
Chr11:65500459	GAACC	0.03	no
**Chr11:65500481**	**GGACU**	**0.07**	**yes**

Supplementary References

1. Chakrabarti, A.M., Haberman, N., Praznik, A., Luscombe, N.M. and Ule, J. (2018) Data Science Issues in Studying Protein–RNA Interactions with CLIP Technologies. *Annu Rev Biomed Data Sci*, **1**, 235–261.
2. König, J., Zarnack, K., Luscombe, N.M. and Ule, J. (2012) Protein-RNA interactions: new genomic technologies and perspectives. *Nat Rev Genet*, **13**, 77-83.
3. Love, M.I., Huber, W. and Anders, S. (2014) Moderated estimation of fold change and dispersion for RNA-seq data with DESeq2. *Genome Biol*, **15**, 550.
4. Krakau, S., Richard, H. and Marsico, A. (2017) PureCLIP: capturing target-specific protein-RNA interaction footprints from single-nucleotide CLIP-seq data. *Genome Biol*, **18**, 240.
5. Anders, S., Reyes, A. and Huber, W. (2012) Detecting differential usage of exons from RNA-seq data. *Genome Res*, **22**, 2008-2017.
6. Frankish, A., Diekhans, M., Ferreira, A.M., Johnson, R., Jungreis, I., Loveland, J., Mudge, J.M., Sisu, C., Wright, J., Armstrong, J. *et al.* (2019) GENCODE reference annotation for the human and mouse genomes. *Nucleic Acids Res*, **47**, D766-D773.
7. Anders, S., Pyl, P.T. and Huber, W. (2015) HTSeq--a Python framework to work with high-throughput sequencing data. *Bioinformatics*, **31**, 166-169.
8. Zhou, Y., Zeng, P., Li, Y.H., Zhang, Z. and Cui, Q. (2016) SRAMP: prediction of mammalian N6-methyladenosine (m6A) sites based on sequence-derived features. *Nucleic Acids Res*, **44**, e91.
9. Chen, Z., Zhao, P., Li, F., Wang, Y., Smith, A.I., Webb, G.I., Akutsu, T., Baggag, A., Bensmail, H. and Song, J. (2020) Comprehensive review and assessment of computational methods for predicting RNA post-transcriptional modification sites from RNA sequences. *Brief Bioinform*, **21**, 1676-1696.
10. Zhang, Y. and Hamada, M. (2018) DeepM6ASeq: prediction and characterization of m6A-containing sequences using deep learning. *BMC Bioinformatics*, **19**, 524.
11. Dominissini, D., Moshitch-Moshkovitz, S., Schwartz, S., Salmon-Divon, M., Ungar, L., Osenberg, S., Cesarkas, K., Jacob-Hirsch, J., Amariglio, N., Kupiec, M. *et al.* (2012) Topology of the human and mouse m6A RNA methylomes revealed by m6A-seq. *Nature*, **485**, 201-206.
12. Meyer, K.D., Saletore, Y., Zumbo, P., Elemento, O., Mason, C.E. and Jaffrey, S.R. (2012) Comprehensive analysis of mRNA methylation reveals enrichment in 3' UTRs and near stop codons. *Cell*, **149**, 1635-1646.
13. Linder, B., Grozhik, A.V., Olarerin-George, A.O., Meydan, C., Mason, C.E. and Jaffrey, S.R. (2015) Single-nucleotide-resolution mapping of m6A and m6Am throughout the transcriptome. *Nat Methods*, **12**, 767-772.
14. Freund, Y. and Schapire, R.E. (1995) In Vitányi, P. (ed.), *Computational Learning Theory. EuroCOLT 1995. Lecture Notes in Computer Science (Lecture Notes in Artificial Intelligence)*. Springer, Berlin, Heidelberg, Vol. 904.
15. Alfaro, E., Gamey, M. and Garcia, N. (2013) adabag: An R Package for Classification with Boosting and Bagging. *J Stat Softw*, **54**, 1-35.
16. Chen, W., Xing, P. and Zou, Q. (2017) Detecting N(6)-methyladenosine sites from RNA transcriptomes using ensemble Support Vector Machines. *Sci Rep*, **7**, 40242.

17. Xiang, S., Liu, K., Yan, Z., Zhang, Y. and Sun, Z. (2016) RNAMethPre: A Web Server for the Prediction and Query of mRNA m6A Sites. *PLoS One*, **11**, e0162707.
18. Chang, C.-C. and Lin, C.-J. (2011) LIBSVM: A library for support vector machines. *ACM Trans Intell Syst Technol*, 27.
19. Liaw, A. and Wiener, M. (2002) Classification and Regression by randomForest. *R News*, **2**, 18-22.
20. Yankova, E., Blackaby, W., Albertella, M., Rak, J., De Braekeleer, E., Tsagkogeorga, G., Pilka, E.S., Aspris, D., Leggate, D., Hendrick, A.G. *et al.* (2021) Small molecule inhibition of METTL3 as a strategy against myeloid leukaemia. *Nature*, **in press**.
21. Boulias, K., Toczydlowska-Socha, D., Hawley, B.R., Liberman, N., Takashima, K., Zaccara, S., Guez, T., Vasseur, J.J., Debart, F., Aravind, L. *et al.* (2019) Identification of the m(6)Am Methyltransferase PCIF1 Reveals the Location and Functions of m(6)Am in the Transcriptome. *Mol Cell*, **75**, 631-643 e638.
22. Koh, C.W.Q., Goh, Y.T. and Goh, W.S.S. (2019) Atlas of quantitative single-base-resolution N(6)-methyl-adenine methylomes. *Nat Commun*, **10**, 5636.
23. Xiao, Y., Wang, Y., Tang, Q., Wei, L., Zhang, X. and Jia, G. (2018) An Elongation- and Ligation-Based qPCR Amplification Method for the Radiolabeling-Free Detection of Locus-Specific N(6)-Methyladenosine Modification. *Angew Chem Int Ed Engl*, **57**, 15995-16000.
24. Liu, N., Parisien, M., Dai, Q., Zheng, G., He, C. and Pan, T. (2013) Probing N6-methyladenosine RNA modification status at single nucleotide resolution in mRNA and long noncoding RNA. *RNA*, **19**, 1848-1856.
